# Natural compounds from herbs and nutraceuticals as glycogen synthase kinase‐3β inhibitors in Alzheimer's disease treatment

**DOI:** 10.1111/cns.14885

**Published:** 2024-08-11

**Authors:** Zheng Zhao, Ye Yuan, Shuang Li, Xiaofeng Wang, Xue Yang

**Affiliations:** ^1^ Department of Emergency Medicine Shengjing Hospital of China Medical University Shenyang Liaoning China; ^2^ Department of Neurosurgery Shengjing Hospital of China Medical University Shenyang Liaoning China; ^3^ Department of Neurology Shengjing Hospital of China Medical University Shenyang Liaoning China

**Keywords:** Alzheimer's disease, biological activity, natural products, phytochemicals, small molecule inhibitor

## Abstract

**Background:**

Alzheimer's disease (AD) pathogenesis is complex. The pathophysiology is not fully understood, and safe and effective treatments are needed. Glycogen synthase kinase 3β (GSK‐3β) mediates AD progression through several signaling pathways. Recently, several studies have found that various natural compounds from herbs and nutraceuticals can significantly improve AD symptoms.

**Aims:**

This review aims to provide a comprehensive summary of the potential neuroprotective impacts of natural compounds as inhibitors of GSK‐3β in the treatment of AD.

**Materials and Methods:**

We conducted a systematic literature search on PubMed, ScienceDirect, Web of Science, and Google Scholar, focusing on in vitro and in vivo studies that investigated natural compounds as inhibitors of GSK‐3β in the treatment of AD.

**Results:**

The mechanism may be related to GSK‐3β activation inhibition to regulate amyloid beta production, tau protein hyperphosphorylation, cell apoptosis, and cellular inflammation. By reviewing recent studies on GSK‐3β inhibition in phytochemicals and AD intervention, flavonoids including oxyphylla A, quercetin, morin, icariin, linarin, genipin, and isoorientin were reported as potent GSK‐3β inhibitors for AD treatment. Polyphenols such as schisandrin B, magnolol, and dieckol have inhibitory effects on GSK‐3β in AD models, including in vivo models. Sulforaphene, ginsenoside Rd, gypenoside XVII, falcarindiol, epibrassinolides, 1,8‐Cineole, and andrographolide are promising GSK‐3β inhibitors.

**Conclusions:**

Natural compounds from herbs and nutraceuticals are potential candidates for AD treatment. They may qualify as derivatives for development as promising compounds that provide enhanced pharmacological characteristics.

## Graphical abstract

GSK‐3β kinase dysregulation aggregates Aβ production and accumulation in Alzheimer's disease (AD). Hyperactive GSK‐3β promotes phosphorylation and formation of toxic tau species. GSK‐3β represents a promising therapeutic target against AD. Nature‐derived phytochemicals inhibiting GSK‐3β activity provided novel therapeutic strategy for AD.

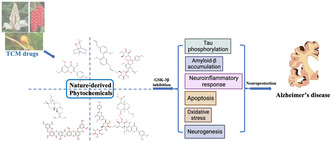

## 1 INTRODUCTION

Alzheimer's disease (AD) is a progressive neurodegenerative disease characterized by progressive memory dysfunction.^1^ As an important factor that threatens the health and quality of life of old individuals, the hazards of AD have caused widespread concern globally.^2^ About 40 million patients worldwide are currently affected by dementia, and if AD is not effectively controlled, by 2050, the number of patients with AD worldwide will be twice or more than the current level.^3^, ^4^ With a large population, China has about 6–8 million patients with AD, and the incidence rate remains high, which has become one of the medical problems endangering the health of the Chinese and the world population.^5^ However, its pathogenesis is still unclear, and AD prevention and treatment still face great challenges.^6^ The typical pathological characteristics of AD are senile plaque formed by deposition of β‐amyloid protein (Aβ) and neurofibrillary tangles (NFTs) formed by tau protein hyperphosphorylation.^7^, ^8^ Inhibiting Aβ deposition and tau protein hyperphosphorylation has become the research focus. Currently, AD treatment is mainly based on medications, and the clinical trials of medications targeting Aβ deposition and tau protein hyperphosphorylation have not made progress, which still cannot prevent AD development.^9^, ^10^ Eventually, AD not only seriously affects the quality of life, but even makes patients with AD lose confidence in life.^11^ Therefore, the search for new therapeutic targets may bring new hope for AD treatment.

Glycogen synthase kinase‐3β (GSK‐3β) is a serine–threonine kinase named for phosphorylated glycogen synthase.^12^ GSK‐3β exerts its biological effects mainly through phosphorylation, intracellular localization, and binding protein regulations. More than 50 kinds of substrates exist mainly, including various structural proteins, metabolic protein enzymes, and transcription factors. The most common are microtubule‐associated tau proteins, β‐catenin, and cyclic adenosine monophosphate (cAMP).^13^ GSK‐3β activity is also regulated via phosphorylation. GSK‐3β activity is inhibited by amino‐terminal serine site (Ser9) phosphorylation, and protein kinases C and A can be phosphorylated at this site to inhibit its activity. Phosphorylation at the tyrosine site (Thr216) can enhance its activity.^14^ GSK‐3β is enriched in the central nervous system, which is involved in various neuronal functions through phosphorylated metabolic enzymes, signaling proteins, structural proteins, and transcription factors, and is associated with various central nervous system diseases, such as AD.^15^ GSK‐3β can regulate multiple signal transduction pathways and is involved in Aβ production, tau protein phosphorylation, neuronal cell apoptosis, and inflammatory reaction, which are closely related to the pathogenic AD changes.^16^ GSK‐3β activity inhibition may become a new therapeutic strategy for AD. Although several chemosynthetic GSK‐3β inhibitors exist in clinical trials for patients with cognitive impairment, none of the compounds are used clinically.^17^

**FIGURE 1:**
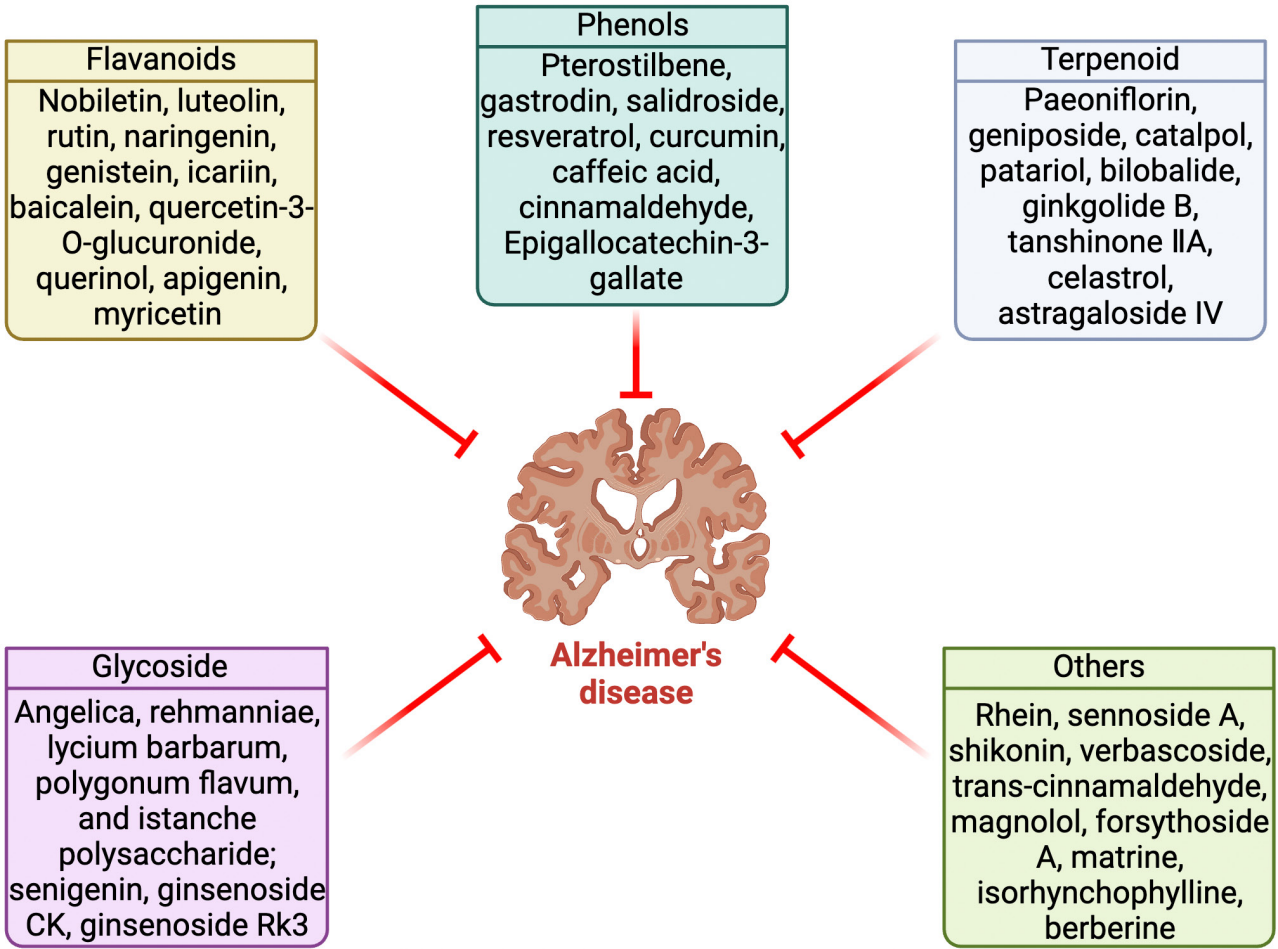
Representative natural compounds that have been demonstrated to exert a therapeutic effect on AD.

Natural compounds from herbs and nutraceuticals are a class of active ingredients with various pharmacological effects.^18^ Natural plants have beneficial anti‐AD effects, and they may prevent or delay AD progression in vivo and in vitro through various mechanisms, such as anti‐inflammation, antioxidative stress, and antiapoptosis (Figure 1).^19^, ^20^ Flavonoids, saponins, phenols, alkaloids, and other natural products have many targets for AD prevention, and the GSK‐3β signaling pathway is one of them.^21^, ^22^ Regarding mechanism, phosphorylation induced by GSK‐3β may be an important factor in phytochemicals to inhibit tau hyperphosphorylation and Aβ deposition.^23^, ^24^

## 2 BIOLOGICAL FUNCTIONS AND GSK‐3β REGULATION

**FIGURE 2:**
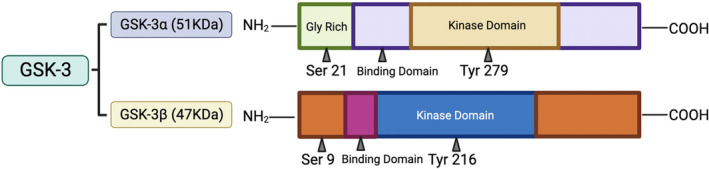
Schematic diagram of mammal GSK‐3 structure. GSK‐3 includes two isoforms of GSK‐3α and GSK‐3β. GSK‐3α contains a glycine‐rich extension at its terminus, accounting for its larger 51 kDa size. Tyr279 phosphorylation located in the T loop (activation site) facilitates substrate phosphorylation by GSK‐3α. GSK‐3β (47 kDa) phosphorylation activation site is at Tyr216, located in the T loop. GSK‐3β phosphorylation at Ser9 in the N‐terminal region leads to inhibition of its kinase activity. The binding domain is specific binding sites for substrates and protein complexes.^23^

GSK‐3 is a multifunctional serine/threonine phosphokinase. GSK‐3 was identified in 1980 and named based on its participation in glycogen metabolism and phosphorylate glycogen synthase. Two GSK‐3 isoforms exist, GSK‐3α (51 kDa) and GSK‐3β (47 kDa).^25^ Although these isoforms share the same substrate, their expression patterns and cellular functions are not identical. GSK‐3 activity is mainly regulated by the phosphorylation of Ser21 residues of GSK‐3α or Ser9 residues of GSK‐3β. When phosphorylated, its activity is inhibited. In addition, its activity can be regulated via subcellular localization, protein complex formation, substrate priming, and proteolysis.^26^ In contrast to the effect of serine residue phosphorylation, tyrosine residues 279 or 216 (Tyr279 of GSK‐3α or Tyr216 of GSK‐3β) phosphorylation enhanced GSK‐3 activity. Currently, phosphorylation of serine residues at position 9 (Ser9 of GSK‐3β) and tyrosine residues at position 216 (Tyr216 of GSK‐3β) are the most widely studied “positive and negative” regulatory mechanisms (Figure 2).

GSK‐3β is involved in regulating several molecular and cellular functions.^27^ In addition to oxidative stress, inflammatory response, and apoptosis, GSK‐3β also affects glycogen synthesis and participates in regulating the cytoskeleton, material transport, and cell cycle at the cellular level.^28^ GSK‐3β was originally identified as a glycogen synthase regulator, which is activated in response to insulin to regulate glycogen synthesis and glucose stabilization.^29^ GSK‐3β can also affect cell polarity and migration by regulating the cytoskeleton and inhibiting GSK‐3β activity in damaged neurons, which is manifested as neuron regeneration and axon elongation.^30^ In addition, GSK‐3β binds to kinesin and can inhibit the transport of membrane vesicles and substances in cells.^31^ During the cell cycle, GSK‐3β participates in the process of cell life by regulating cyclin activity.^32^

**FIGURE 3:**
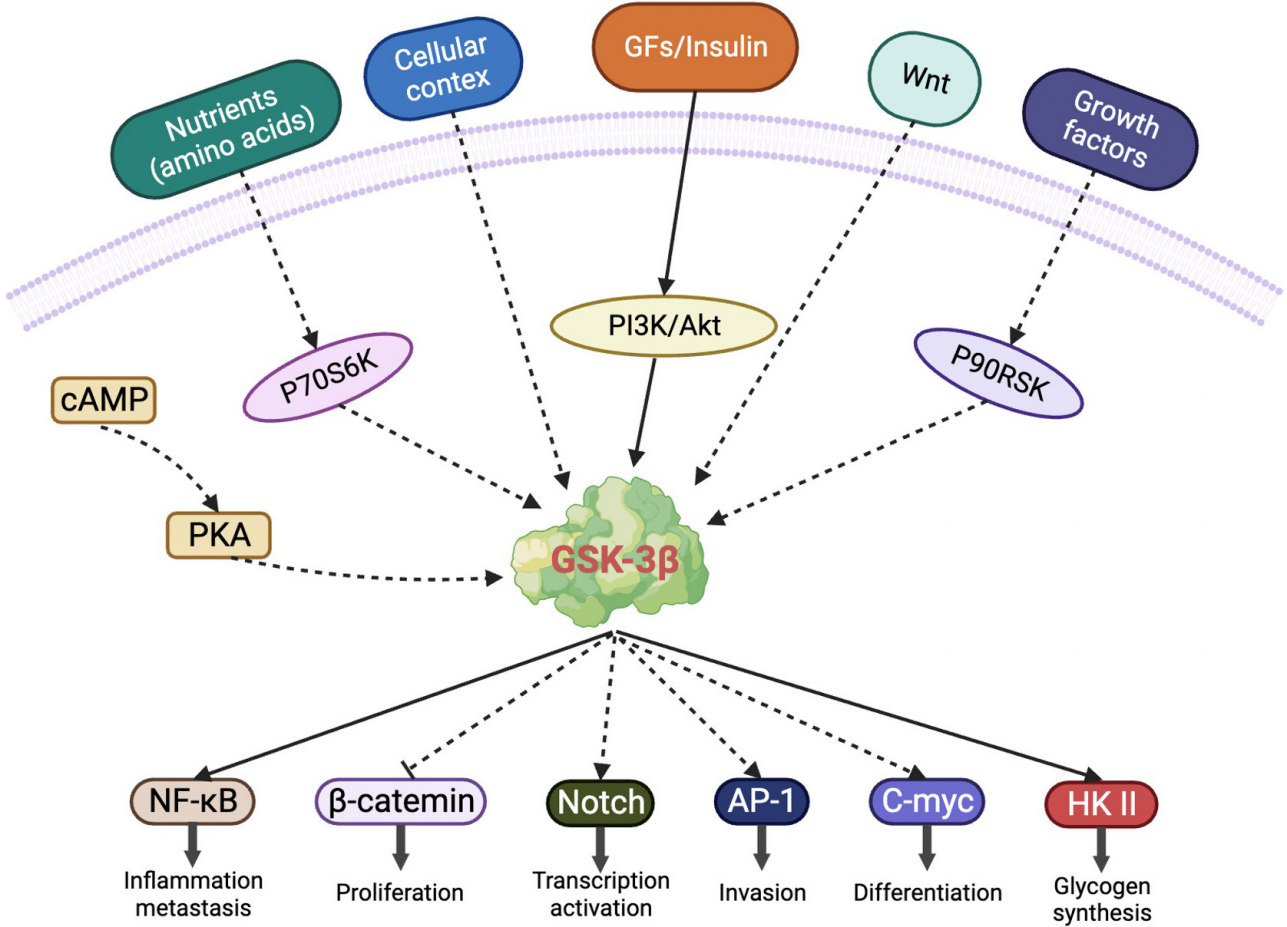
Schematic representation of GSK‐3β signaling pathways and activity regulation.^191^

GSK‐3β is the main regulatory molecule of multiple signaling pathways, involved in glucose metabolism, apoptosis, and senescence, and mediates the process of some diseases.^33^ GSK‐3β activity is negatively regulated by phosphoinositide 3‐kinase (PI3K), Wnt/β‐catenin, Reelin, Hedgehog, Notch, and other signaling pathways, which have important effects on cell growth and metabolism.^34^ (i) Tropomyosin receptor kinase (Trk)‐PI3K‐protein kinase b (Akt) pathway: activating the catalytic subunit of PI3K converts phosphatidylinositol‐4, 5‐diphosphate to phosphatidylinositol‐3, 4, 5‐triphosphate, which in turn phosphorylates Akt, phosphorylates GSK‐3β at Ser9, and downregulates its activity.^35^ (ii) Notch pathway: GSK‐3β is a kinase that regulates notch signaling. The Trk‐PI3k‐AKT pathway acts to gain control of the notch signaling response by inhibiting GSK‐3β.^36^ (iii) The canonical Wnt pathway is β‐catenin dependent. When Wnt signaling is activated, extracellular Wnt proteins bind to frizzled and low‐density lipoprotein 5/6 receptors on the cell surface to activate disheveled proteins, thereby inactivating GSK‐3β and inhibiting β‐catenin degradation.^37^ (iv) Hedgehog pathway: Similar to the Wnt pathway, the hedgehog pathway relies on Cubitus interruptus protein to regulate its target cells and control gene transcription and expression.^38^ (v) Reelin pathway: Reelin glycoprotein binding to very low‐density lipoprotein receptor and apolipoprotein E receptor 2 leads to tyrosine kinase family/fyn‐kinase activation and Dab1‐transductant protein phosphorylation, which in turn activates the Trk‐PI3K‐Akt pathway. Brain‐derived neurotrophic factor (BDNF) (blood–brain barrier [BBB]) inhibits GSK‐3β activity by binding to Trk receptors and activating PI3K and Akt.^39^ (vi) Nuclear factor kappa B (NF‐κB) activation can inhibit GSK‐3β activity and affect the physiological and pathological processes of cells by regulating GSK‐3β substrate phosphorylation and stability. NF‐κB can also indirectly affect GSK‐3β function by regulating key molecules in the Wnt/β–catenin signaling pathway, such as β‐catenin and axin. In contrast, GSK‐3β can affect NF‐κB activity by phosphorylating and regulating key proteins in the NF‐κB activation pathway, such as inhibitors of κB (IκB) and IκB kinase.^40^ Additionally, GSK‐3β can also regulate NF‐κB‐mediated gene transcription and inflammatory response.^41^ (vii) Activator protein 1 (AP‐1) is a transcription factor complex composed of c‐Jun, c‐Fos, and other members. AP‐1 is involved in regulating biological processes, such as cell cycle, proliferation, differentiation, and inflammation.^42^ GSK‐3β can affect AP‐1 activity by regulating the expression and stability of AP‐1 members. GSK‐3β can affect AP‐1 activity by phosphorylating and regulating transcription and translation of members, such as c‐Jun and c‐Fos. However, GSK‐3β can also affect AP‐1 function by regulating the gene transcription and cell signaling pathways mediated by AP‐1 (Figure 3).^43^

Since GSK‐3β is concentrated in the central nervous system, GSK‐3β activity disorder can lead to the occurrence and development of various nervous system diseases, such as stroke, neurodegenerative diseases (AD), epilepsy, and spinal cord injury.^44^ Recently, with the deepening of research on the relationship between GSK‐3β and the occurrence and development of various diseases, the research on targeting GSK‐3β in treating various nervous system diseases has become an important topic.^45^

## 3 GSK‐3β AND AD

**FIGURE 4:**
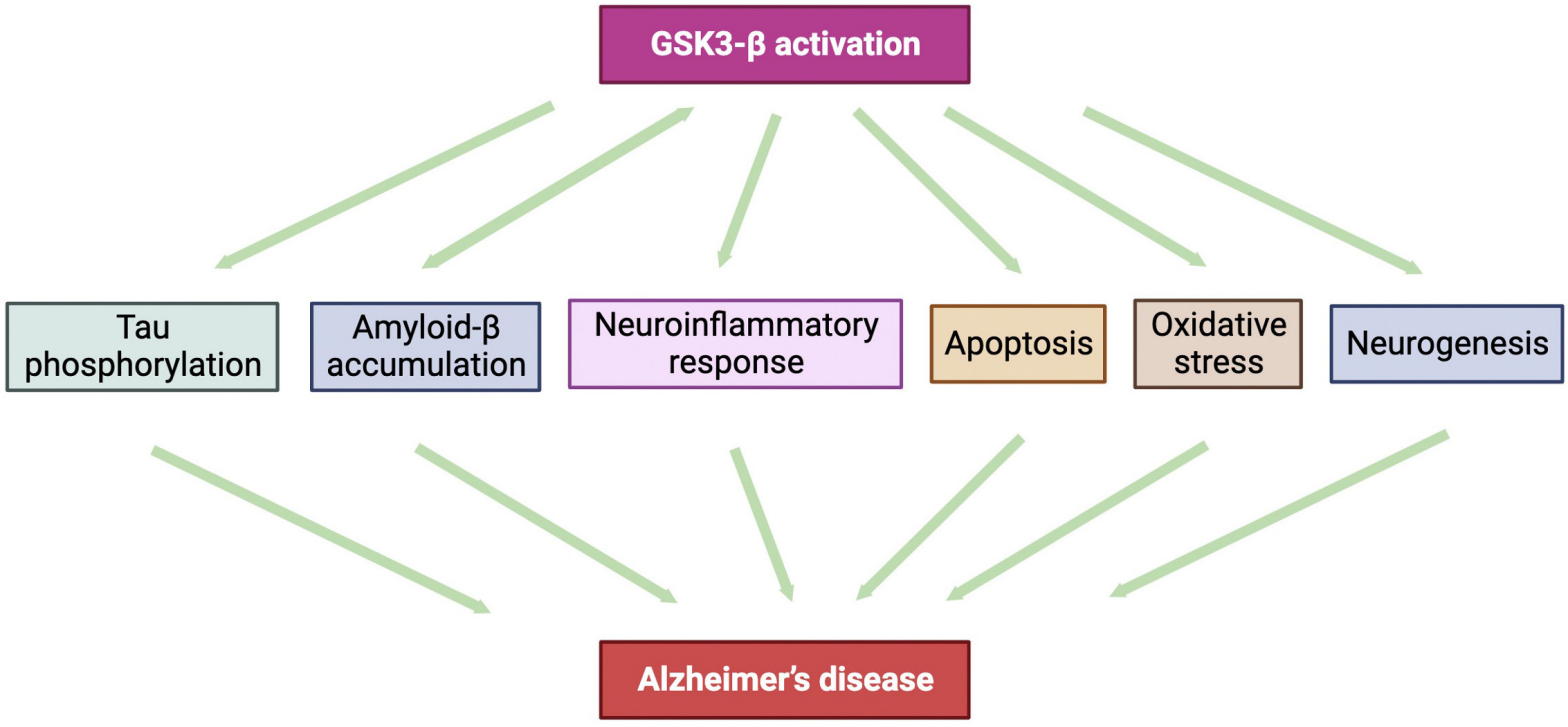
Role of GSK‐3β signaling in AD pathology. Schematic representation of the potential involvement of GSK‐3β in different aspects and pathways relevant to AD neuropathology onset and development. GSK‐3β activation contributes to neurodegeneration by directly promoting tau hyperphosphorylation. Hyperphosphorylated tau dissociates from the microtubules, leading to impaired axonal transport and NFT formation. GSK‐3β promotes amyloid production and accumulation, which induces apoptosis and neuronal damage in AD. GSK‐3β also displays proinflammatory functions. GSK‐3β promotes inflammatory molecule production. Additionally, GSK‐3β plays a critical role in regulating hippocampal neurogenesis, an important process that supports specific forms of learning and memory and is affected in AD pathology.^192^

GSK‐3β expression in the central nervous system is extremely high, especially in the hippocampus, which is a key brain area involved in learning, memory, and emotion regulation in the brain, and is involved in integrating external information into the central transduction.^46^ GSK‐3β is required for early development in the hippocampus and plays a key role in establishing and maintaining neuronal polarity.^47^ While GSK‐3β is ubiquitously distributed in axons and dendrites, inactive GSK‐3β is mainly localized in axon terminals.^48^ The biological basis of learning and memory is synaptic plasticity, which is quantified as long‐term potentiation (LTP) in the hippocampus.^49^ GSK‐3β is a multifunctional kinase whose alterations lead to structural changes in neural networks, developmental abnormalities, dysregulated signaling pathways, and altered brain plasticity. GSK‐3β activity is increased in patients with mild cognitive impairment,^50^ and GSK‐3β can inhibit neuronal axon regeneration in the central nervous system.^51^ GSK‐3β in the dentate gyrus can impair fear memory and synaptic plasticity.^52^ GSK‐3β inhibition is required for LTP induction, and GSK‐3β activation inhibits LTP and reduces synaptic transmission and release of the presynaptic neurotransmitter glutamate.^53^ Furthermore, GSK‐3β can participate in AD pathogenesis by regulating multiple intracellular signaling pathways, interfering with Aβ deposition, tau protein hyperphosphorylation, nerve cell apoptosis, and neuroinflammatory response, and other mechanisms (Figure 4).^54^

### 3.1 GSK‐3β and Aβ deposition in AD

Aβ deposition is the most important pathological feature in AD pathogenesis. Increased GSK‐3β activity can promote Aβ production and deposition, and abnormal Aβ aggregation can, in turn, increase GSK‐3β activity, thereby aggravating pathological AD development.^55^ Aβ is derived from β‐amyloid precursor protein (APP) hydrolysis. GSK‐3β plays an important role in regulating APP and its catabolic enzymes α, β, and γ.^56^ In the nonamyloid proteolytic pathway, APP is sequentially hydrolyzed by α and γ secretases. GSK‐3β can downregulate α‐secretase complex activity and inhibit the nonamyloid proteolysis of APP by inhibiting metalloproteinase (ADAM) activity.^57^ In the amyloidogenic pathway, APP is first cleaved by β secretase, with subsequent Aβ_40_ or Aβ_42_ release in response to γ secretase. β secretase (BACE1) cleaves APP, and then presenilin‐1 (PS1), the APP substrate, regulates γ‐secretase activity and releases Aβ_40_ or Aβ_42_ to promote Aβ generation in the brain.^58^ GSK‐3β can also induce BACE1 gene expression by upregulating NF‐κB signaling. The BACE1 promoter region contains two functional NF‐κB binding sites. GSK‐3β can upregulate BACE1 gene expression through the NF‐κB/p65 *cis*‐acting element on the BACE1 gene promoter. When GSK‐3β activation is inhibited, the transcription and BACE1 gene expression levels also decrease, APP hydrolysis level mediated by BACE1 decreases, Aβ level also decreases, and memory deficits of rats are improved.^59^ Lithium, a GSK‐3β inhibitor, can protect rat hippocampal neurons from Aβ damage and cerebral nerve function by inhibiting GSK‐3β.^60^, ^61^ Aβ can also activate GSK‐3β and cyclin‐dependent kinase 5 (CDK5) and then affect microtubule‐associated protein tau hyperphosphorylation.^62^ Therefore, GSK‐3β activation level is particularly critical in Aβ formation and accumulation, and GSK‐3β inhibition activation may play a therapeutic role in AD by reducing Aβ formation and accumulation.

### 3.2 GSK‐3β and tau protein hyperphosphorylation in AD

Tau protein is a microtubule‐associated protein in the central nervous system that stabilizes neuronal cell structure and axonal transport. Tau protein activity is regulated by phosphorylation and dephosphorylation. Tau protein phosphorylation is controlled by various protein kinases and phosphatases.^63^ Abnormal tau accumulation interferes with synaptic function, impeding axonal transport, affecting neurotrophic function, and ultimately leading to neuronal death by triggering apoptotic signals.^64^ GSK‐3β is the key kinase that promotes tau protein phosphorylation and is closely related to tau protein phosphorylation at multiple sites.^65^ GSK‐3β is the main kinase involved in abnormal tau protein phosphorylation in vivo.^66^ Several amino acid residues on tau protein are affected by GSK‐3β phosphorylation,^67^ and GSK‐3β phosphorylation at the main site can lead to tau protein phosphorylation.^68^ Additionally, protein phosphatase 2A (PP2A) is a pleiotropic phosphatase that dephosphorylates tau protein in the brain. The imbalance between PP2A and GSK‐3β is also one of the reasons for tau protein hyperphosphorylation.^69^ Treating AD model mice with GSK‐3β inhibitors can improve tau protein hyperphosphorylation and NFT formation in vivo.^70^ Inhibiting tau protein hyperphosphorylation by regulating GSK‐3β activation may be one of the potential strategies for AD treatment.

### 3.3 GSK‐3β and neuroinflammatory response in AD

Neuroinflammatory response is also one of the important mechanisms in AD pathogenesis, and GSK‐3β is a key kinase in inflammatory response.^71^ GSK‐3β is involved in regulating NF‐κB, cAMP response element binding protein (CREB), and other downstream substrates, which are closely related to inflammatory response and promote various proinflammatory cytokine productions. When GSK‐3β is activated, GSK‐3β can promote REL‐A (p65) and B‐cell lymphoma 3 protein (BCL‐3) phosphorylation on NF‐κB. The phosphorylated BCL‐3 binds to the DNA of NF‐κB1 (p50) and NF‐κB2 (p52) on NF‐κB to form a trimer, which induces cell inflammatory reaction.^72^, ^73^ Interleukin‐10 (IL‐10) is one of the main anti‐inflammatory factors. Its effect inhibits the expressions of cyclooxygenase‐2, IL‐1β, and IL‐8, thereby controlling the inflammatory response. GSK‐3β can reduce CREB nuclear translocation, inhibit IL‐10 expression, and aggravate inflammatory response through the PI3K/AKT/GSK‐3 pathway.^74^ GSK‐3β inhibitor can attenuate GSK‐3β and GSK‐3β‐Tyr16 activities in AD rat models, which can reduce the inflammatory response and protect neurons, suggesting that GSK‐3β is involved in neuroinflammatory response occurrence.^75^

### 3.4 GSK‐3β and apoptosis in AD

GSK‐3β can regulate neuronal apoptosis through autophosphorylation. Inhibiting GSK‐3β phosphorylation can prevent neuronal apoptosis and promote neural stem cell proliferation and differentiation.^76^ In the brain of AD rats, GSK‐3β can induce apoptosis by activating the apoptosis‐related protein kinases caspase family and Bax apoptosis gene.^77^ In addition, GSK‐3β is involved in the neuroprotection mediated by histamine H3 receptor. Histamine H3 receptor phosphorylation can inactivate GSK‐3β, promote antiapoptotic gene Bcl‐2 expression, and protect cortical neurons from neurotoxic damage. This suggests that a negative regulatory relationship between GSK‐3β and Bcl‐2 expressions may exist.^78^ These studies suggest that inhibiting GSK‐3β expression, enzyme activity, or its phosphorylation modification process may play an important role in alleviating neuronal apoptosis and promoting neuronal proliferation. Inhibiting the pathological mechanism of nerve cell loss in a brain with AD may be a therapeutic target. However, studies have found that increased GSK‐3β expression and activity can induce neurogenesis. Jurado et al.^79^ found that high GSK‐3β expression can promote the proliferation of mouse neural stem cells and improve the spatial memory ability of mice. Hur et al.^80^ demonstrated that GSK‐3β was involved in differentiating newly formed nerve cells, proliferating neural stem cells, and the growth of neural axons. Therefore, the view that GSK‐3β regulates neuronal apoptosis and proliferation is still controversial; therefore, whether GSK‐3β regulates neuronal apoptosis or neuronal proliferation is unclear.

### 3.5 GSK‐3β and oxidative stress in AD

Besides the involvement of main pathological mechanisms, such as Aβ deposition and tau protein hyperphosphorylation, oxidative stress plays an important role in AD occurrence and development.^81^ Reactive oxygen species (ROS) are a kind of oxygen‐containing substances with active chemistry and strong oxidation ability in organisms.^82^ They can destroy the structure and function of biofilms and other polymers, promote aging, and cause extensive damage to the body.^83^ ROS have toxic effects on most cells and can balance ROS levels in the body by degrading reactive oxygen and nitrogen radicals.^84^ Excessive ROS accumulation, which is called oxidative stress, can attack the body. With the increase in age, during the aging process of brain tissue, unsaturated fatty acids on the cell membrane of neurons are oxidized, and a large amount of ROS is produced. Compared with other tissues and organs, the brain is more vulnerable to attack under oxidative stress.^85^ ROS‐inhibited PI3K can indirectly activate GSK‐3β, and the activated GSK‐3β can increase tau phosphorylation, leading to the NFT formation and destabilization and, ultimately, microtubule depolymerization.^86^, ^87^

### 3.6 GSK‐3β and neurogenesis in AD

Neurogenesis is essential for hippocampal function and hippocampal‐dependent memory.^88^ GSK‐3β is one of the most important regulators of adult hippocampal neurogenesis, and its overexpression impairs adult neurogenesis and leads to a reduced number of neuronal clusters in the hippocampal dentate gyrus.^89^ This impaired neurogenesis may also be related to microglia activation.^90^ GSK‐3β phosphorylation can cause β‐catenin accumulation in the cytoplasm and promote β‐catenin entry into the nucleus, forming a complex with the T‐cell factor/lymphoid‐enhancing factor protein, thereby activating the transcription of corresponding genes and effectively regulating neural progenitor cell growth and development and regulating neuronal cell growth, proliferation, differentiation, and apoptosis.^91^ GSK‐3β activation inhibition may promote neurogenesis and play a protective role in AD‐related neurons.

### 3.7 GSK‐3β affects AD by regulating signaling pathways

Besides its role in PI3K/Akt and Wnt/β‐catenin signaling pathways, GSK‐3β is also involved in AD pathological mechanisms through other signaling pathways. The mitogen‐activated protein kinase (MAPK) superfamily has several distinct signaling pathways, such as extracellular signal‐regulated kinases, p38, and c‐Jun N‐terminal kinases (JNK).^92^ MAPK can transmit various cellular response signals to environmental stimuli, such as inflammatory cytokines, and environmental stress signals can activate JNK, induce stress responses, and lead to cell growth arrest and apoptosis.^93^ The three MAPK pathways ERK, p38, and JNK are activated in AD pathogenesis.^94^ JNK exerts an important role in tau protein hyperphosphorylation and can interact with various tau protein kinases, among which GSK‐3β is an important member of JNK phosphorylated tau protein. In the brain of insulin gene knockout mice, JNK and GSK‐3β are phosphorylated simultaneously with tau protein hyperphosphorylation, indicating that the lack of insulin stimulation in the brain can cause JNK and GSK‐3β activation and induce tau protein phosphorylation, suggesting that JNK and GSK‐3β play a synergistic regulatory role in the process of tau protein phosphorylation.^95^

BDNF is highly expressed in the hippocampus of the mammalian brain, which is a brain structure related to spatial learning and memory.^96^ BDNF expression is regulated by CREB. GSK‐3β activation can lead to CREB‐targeted gene expression (such as BDNF) downregulation, while GSK‐3β inhibition can improve cognitive impairment.^97^ Therefore, GSK‐3β may be involved in AD occurrence and development by affecting CREB and BDNF.

## 4 GSK‐3β INHIBITORS AND AD

GSK‐3β participates in AD occurrence and development through multiple signaling pathways. Studies on GSK‐3β as a target to prevent and treat AD exist, especially on GSK‐3β inhibitors. Compounds acting on this target are mainly adenosine triphosphate (ATP) and non‐ATP competitive inhibitors.^98^

In preclinical studies, selective GSK‐3β inhibitors improve memory,^99^ mood,^100^ and emotional processing.^101^ They also reduce inflammation and provide beneficial effects on Aβ and tau pathology in AD animal models.^102^, ^103^ Thiadiazolidinones (TDZD, IC_50_ = 2 μmol/L) is the first non‐ATP competitive inhibitor. In the experiment on whether GSK‐3β inhibitor can reduce tau protein phosphorylation, neuronal apoptosis, and improve memory in APP and tau protein coexpressed mice, it was observed that TDZD can block GSK‐3β involved in neurodegenerative disease‐related proapoptotic signaling cascade, thereby improving cognitive function^104^, ^105^ and reversing hyperphosphorylated tau protein expression.^106^ Tideglusib (NP‐12), a non‐ATP‐competitive GSK‐3β inhibitor, significantly reduced p‐tau, Aβ deposition, senile plaque‐associated astrocyte proliferation, and hippocampal CA1 cell apoptosis while protecting neurons in the entorhinal region and improving memory impairment in mice.^107^ N‐(1,3‐benzodioxol‐5‐yl)‐2‐(5‐chloro‐2‐methoxy [phenylsulfonyl] aniline) acetamide (LX2343) (IC_50_, 1.84 ± 0.07 μmol/L) effectively inhibited tau protein phosphorylation in nerve cells and improved cognitive dysfunction in AD rat models by inhibiting oxidative stress‐induced neuronal apoptosis and tau protein lesions.^108^ Lithium is a non‐ATP‐competitive and selective GSK‐3β inhibitor, which may inhibit GSK‐3β by removing potassium ions or magnesium ions from binding to the enzyme.^109^ In addition, lithium reduced Aβ production by downregulating tau phosphorylation.^110^ GSK‐3β activity and tau protein phosphorylation level in transgenic mice treated with lithium for 30 days were significantly inhibited.^111^ Lithium inhibited tau phosphorylation by inhibiting GSK‐3β in animal models.^112^

ATP‐competitive inhibitors indirubin, hymenialdisin, and SB216763 can inhibit GSK‐3β enzyme activity, alleviate Aβ deposition and tau hyperphosphorylation‐associated neuropathological changes, and improve cognitive impairment in AD animal models.^113^, ^114^ Fukunaga et al. synthesized 2‐(2‐phenylmorpholin‐4‐yl) pyrimidin‐4(3H) compounds and examined their inhibitory activity against GSK‐3β. Compound 21 had potent in vitro GSK‐3β inhibitory activity with favorable in vitro pharmacokinetic profiles, showing a significant tau phosphorylation reduction and excellent pharmacokinetic profiles in AD mice after oral administration.^87^ SB415286 is an ATP‐competitive inhibitor. SB415286 competes with ATP for binding to GSK‐3β ATP‐binding site, a serine/threonine protein kinase, to effectively inhibit the kinase activity of GSK‐3 and interfere with its downstream signaling pathways. SB415286 has been studied for its potential therapeutic effects in AD by inhibiting GSK‐3β activity. By blocking GSK‐3β activity, SB415286 may facilitate tau protein hyperphosphorylation reduction and Aβ peptide production, potentially slowing AD pathology progression.^115^ AR‐A014418, as an ATP‐competitive GSK‐3β inhibitor, inhibited GSK‐3β‐specific tau phosphorylation at Ser‐396 in cells stably expressing human quadruple‐repeat tau protein. Additionally, AR‐A014418 inhibited Aβ‐mediated neurodegeneration in hippocampal slices. Therefore, AR‐A014418 may have important application value in elucidating the role of GSK‐3β in cell signaling and possibly in AD treatment.^116^ CHIR‐99021 specifically targets the ATP‐binding site of GSK‐3β and competitively inhibits its activity.^67^ CHIR‐99021 reduces tau phosphorylation, decreases tau levels in human glutamatergic neurons, and may enhance the ability of neurons to antagonize tau pathology.^117^ Alsterpaullone competes with ATP for binding to GSK‐3β. Alsterpaullone inhibited tau phosphorylation in vivo at sites which are typically phosphorylated by GSK‐3β in AD.^118^

As a potent and highly selective GSK‐3β inhibitor, 1,3,4‐oxadiazole derivative not only exhibited high selectivity and potent inhibitory activity against GSK‐3β, but also exhibited favorable pharmacokinetic profiles, including favorable BBB penetration. Among these compounds, identified by Saitoh et al.,^119^ (S)‐9b and (S)‐9c orally administered to mice significantly inhibited cold water stress‐induced tau hyperphosphorylation in the brain of mice and improved cognitive behavior of mice and may be potential drugs for AD treatment. Onishi et al. conducted a study on another novel 1,3,4‐oxo‐derived GSK‐3β inhibitor 2‐methyl‐5‐(3–4‐[(S)‐methyl sulfinyl] phenyl‐1‐benzofuran‐5‐yl)‐1,3,4‐oxadiazole (MMBO), and the result indicated that MMBO significantly inhibited tau phosphorylation in primary cultured neural cells and normal mouse brain. An in vivo study also confirmed that MMBO significantly reduced tau phosphorylation at GSK‐3β in the hippocampus of transgenic AD mice. In the behavioral assessment, MMBO ameliorated memory and cognitive deficits in the Y‐maze and novel object recognition tests in the transgenic AD mouse model. These results suggested that pharmacological GSK‐3β inhibition improves behavioral disorders in an AD mouse model by inhibiting tau phosphorylation, and MMBO may be beneficial for AD treatment.^120^ Lu et al. modified the structure of 4‐methylquinoline, synthesized new compounds, and evaluated their neuroprotective activity. The results suggested that compound 3 showed nanomolar protection and GSK‐3β enzyme activity inhibition in Aβ‐induced MC65 cells. Using a normal mouse model, the researchers investigated the distribution of compound 3 in different tissues and its possible toxic effects on exercise. Compound 3 did not cause a decrease in exercise activity in mice, and no significant liver transaminase inhibition was observed, suggesting that the long‐term use of this compound in AD mouse models is safe (Table 1).^121^

**TABLE 1:** GSK‐3β inhibitors with their therapeutic application in Alzheimer's disease.

Type of inhibition	Inhibitor	Pharmacological effect	Structure	References
Non‐ATP‐competitive	Thiadiazolidinones	Reduce tau phosphorylation, neuronal apoptosis	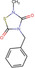	Koehler et al.,^104^ Liang et al.^105^
	Tideglusib	Reduce p‐tau, Aβ deposition, senile plaque‐associated astrocyte proliferation, and hippocampal CA1 cell apoptosis	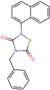	Serenó et al.^107^
	LX2343	Inhibit the phosphorylation of tau protein in nerve cells	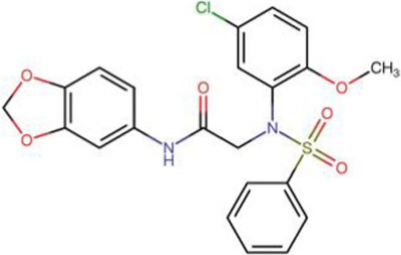	Guo et al.^108^
ATP‐competitive	Indirubin	Alleviate Aβ deposition and tau hyperphosphorylation	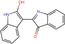	Ding et al.^113^
	SB216763	Alleviate Aβ deposition and tau hyperphosphorylation	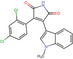	Lin et al.^114^
	Compound 21	Reduce tau phosphorylation	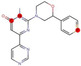	Fukunaga et al.^87^
	SB415286	Reduce the hyperphosphorylation of tau protein and the production of Aβ peptides	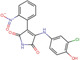	Wang et al.^115^
	AR‐A014418	Inhibit GSK‐3β specific tau phosphorylation at Ser‐396, attenuate Aβ‐mediated neurodegeneration	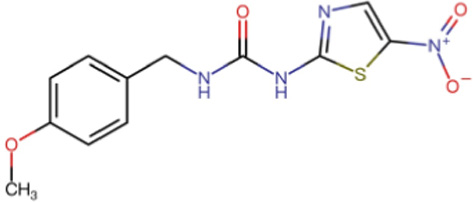	Bhat et al.^116^
	CHIR‐99021	Reduce tau phosphorylation and decrease tau levels	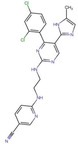	Cheng et al.^117^
	Alsterpaullone	Inhibit the phosphorylation of tau	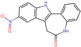	Leost et al.^118^
	(S)‐9b and (S)‐9c	Inhibit tau hyperphosphorylation	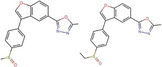	Saitoh et al.^119^

	MMBO	Inhibit tau phosphorylation	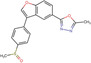	Onishi et al.^120^
Compound 3	Reduce the hyperphosphorylation of tau protein and the production of Aβ peptides	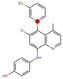	Lu et al.^121^

Abbreviations : LX2343, N‐(1,3‐benzodioxol‐5‐yl)‐2‐[5‐chloro‐2‐methoxy (phenylsulfonyl) anilino] acetamide; MMBO, 2‐methyl‐5‐(3–4‐[(S)‐methyl sulfinyl] phenyl‐1‐benzofuran‐5‐yl)‐1,3,4‐oxadiazole.

Of the known GSK‐3β inhibitors, lithium carbonate and tideglusib are the most intensively studied in clinical trials (Table 2), and these clinical trials were designed to evaluate the potential and anti‐AD properties of lithium carbonate and tideglusib as GSK‐3β inhibitors for AD prevention in older adults with mild cognitive impairment and at risk for dementia.^122^, ^123^ Tideglusib is a potent, selective, and irreversible non‐ATP‐competitive GSK‐3β inhibitor with neuroprotective activity.^124^, ^125^ However, phase II clinical trials confirmed that tideglusib had no clinical benefit compared with placebo, even though the drug was well tolerated.^126^, ^127^

**TABLE 2:** Clinical investigation of GSK‐3β inhibitors as the future therapy of Alzheimer's disease.

NCT number	Intervention	Start date	Phase	Study type/intervention model	Number of participants	Title	Status
NCT02601859	Lithium carbonate	August 2016	NA	Interventional/single group assignment	11	Evaluation of lithium as a glycogen synthase kinase‐3 inhibitor in mild cognitive impairment	Completed
NCT03185208	Lithium carbonate/placebo	September 2017	IV	Interventional/parallel assignment	80	Evaluation of brain and cognitive changes in older adults with MCI taking lithium to prevent Alzheimer type dementia	Recruiting
NCT00948259	Tideglusib/placebo	December 2008	I, II	Interventional/parallel assignment	30	Phase IIa 20‐week double‐blind, Placebo‐controlled, randomized, escalating dose study to evaluate the safety and tolerability of four oral doses of NP031112, a novel GSK3 inhibitor, in mild‐to‐moderate Alzheimer's disease patients with stable anticholinesterase treatment	Completed
NCT01350362	Tideglusib/placebo	April 2011	II	Interventional/parallel assignment	306	A multicenter, randomized, double‐blind, placebo‐controlled, four‐arm, 26‐week parallel group study to evaluate efficacy, safety, and tolerability of two oral doses and two regimes of tideglusib versus placebo in mild‐to‐moderate AD patients	Completed

## 5 DRUG DISCOVERY FOR AD

Although synthetic chemical drugs targeted for AD symptoms have been developed, there is still a demand for effective treatments due to the lack of both efficacy and safety in currently available therapeutic agents. Drugs approved by the U.S. Food and Drug Administration for AD treatment are donepezil, tacrine, galantamine, and cabaladin (rivastigmine).^128^, ^129^ These drugs are cholinesterase (AchE) inhibitors, which can alleviate ACh deficiency caused by damage to the cerebral cortex and basal forebrain cholinergic pathways, thereby improving cognitive function.^130^ The non‐competitive *N*‐methyl‐d‐aspartic acid (NMDA) receptor antagonist memantine was the first drug approved for moderate–severe AD.^131^ AchE inhibitors with NMDA receptor antagonists are used for severe symptoms, and donepezil with memantine capsule was used clinically in 2014. However, drugs that act on the histaminergic, gamma‐aminobutyric, and 5‐hydroxytryptamine systems are still being developed.^132^ Although studies on AchE inhibitors and NMDA receptor antagonists are in depth, the effect of these drugs on patients with severe AD is still not optimistic even if bradycardia, syncope, and severe gastrointestinal adverse reactions, including esophageal rupture caused by drugs (especially AchE inhibitors), are ignored.^133^ Therefore, studying targeted drugs may become a new research center in AD treatment.

In animal studies of AD, nonsteroidal anti‐inflammatory drugs (NSAIDs) delay or even prevent AD. However, the risk of heart and stomach bleeding must be considered, limiting NSAID application.^134^ In addition, improved cognitive impairment was found in APPswe/PS1E9 transgenic mice treated with valproic acid.^135^ In epithelial fibroblasts, sodium arsenite enhances DNA base excision repair, showing its potential as an antidementia agent. However, this effect is strongly dose dependent, and arsenate, a well‐known neurotoxic heavy metal, interferes with learning and affects behavioral parameters.^136^, ^137^ In addition, at high doses, sodium arsenate can promote ROS formation, resulting in DNA or protein damage, thereby promoting cocarcinogenic and protumor capabilities.^138^

GSK‐3β plays an important role in Aβ formation and tau protein hyperphosphorylation in neuronal and non‐neuronal cells.^139^ GSK‐3β inhibitors may reduce Aβ formation, inhibit tau protein hyperphosphorylation, participate in regulating other AD pathogenic mechanisms, reduce neuronal damage, and improve AD.^17^ Phytochemicals have become an important topic in the treatment of chronic diseases owing to their multiple targets, low toxicity, and wide therapeutic range.^140^, ^141^ Phytochemicals with GSK‐3β inhibitory effect, as drug leads, pave the way for developing improved drugs to prevent AD or delay its progression.

## 6 NATURAL COMPOUNDS FROM HERBS AND NUTRACEUTICALS AS GSK‐3β INHIBITORS

**FIGURE 5:**
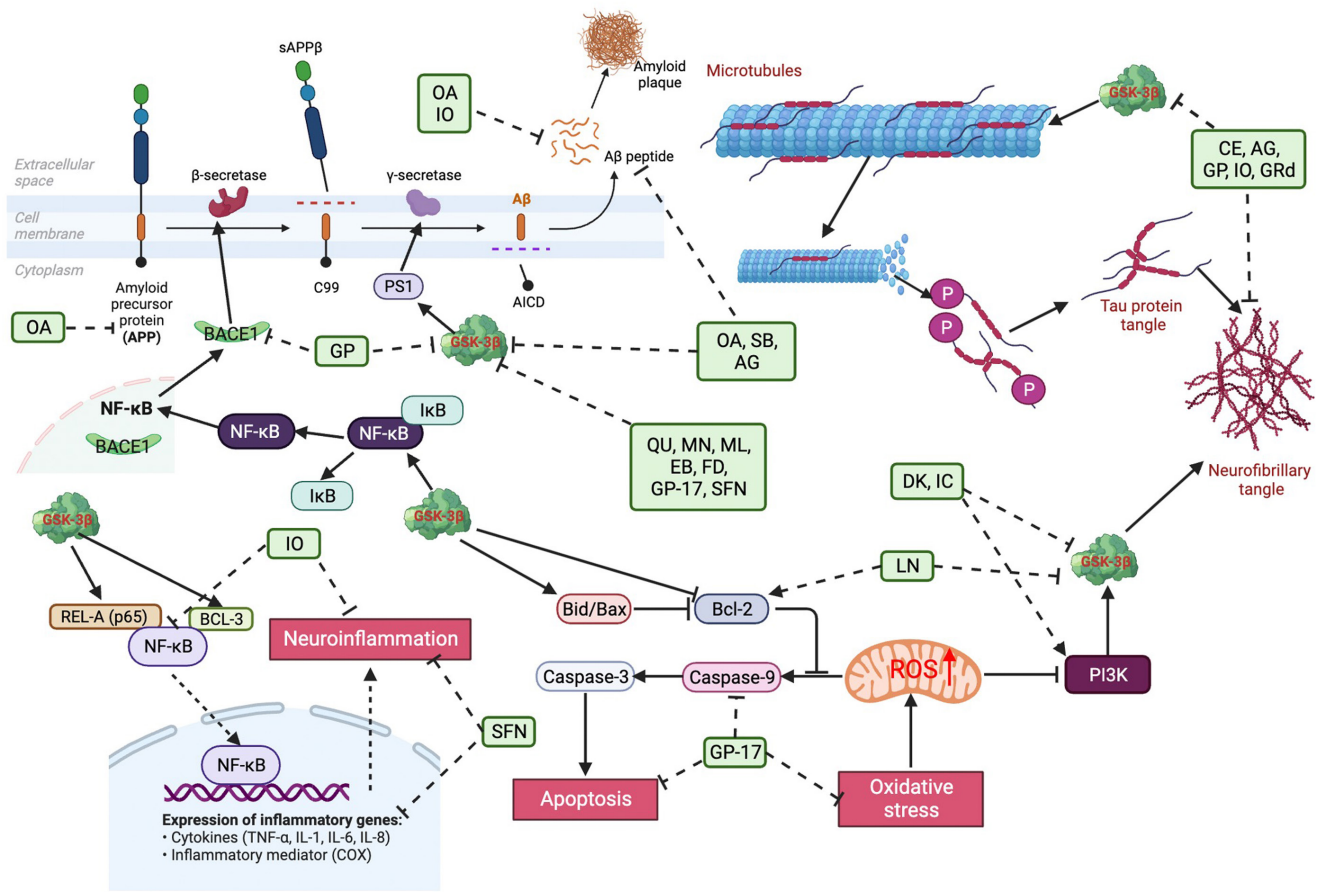
Schematic diagram of phytochemicals inhibiting GSK‐3β activity in AD treatment. Aβ is generated from APP by the sequential cleavage of β and γ secretases. Phytochemicals could inhibit GSK‐3 activity, modulate APP processing, and downregulate β and γ secretases in the Aβ generation process. Tau phosphorylation at crucial sites separates tau from microtubules, breaks down microtubules, leads to the accumulation of tau aggregated into paired helical filaments, and eventually supports NFT establishment. Tau phosphorylation by GSK‐3 is involved in many tau sites, especially in the proline‐rich and COOH‐terminus regions. Phytochemicals could increase pSer9‐GSK‐3β levels, inhibiting tau protein phosphorylation and NFT formation. Some phytochemicals exert anti‐inflammatory, antioxidant, and antiapoptotic activities in AD models by inhibiting GSK‐3β activity. AG, andrographolide; CE, 1,8‐cineole; DK, dieckol; EB, epibrassinolides; FD, falcarindiol; GP, genipin; GRd, ginsenoside Rd; IC, icariin; IO, isoorientin; LN, linarin; ML, magnolol; MN, morin; OA, oxyphylla A; QU, quercetin; SB, schisandrin B; SFN, sulforaphene; Aβ, amyloid beta; APP, amyloid precursor protein; NFT, neurofibrillary tangle; GSK‐3, glycogen synthase kinase 3; AD, Alzheimer's disease; GSK‐3β, glycogen synthase kinase 3β.

The origin, classification, and pharmacological effects of discussed phytochemicals are presented in Table 3, and an overview of GSK‐3β inhibition by phytochemicals is depicted in Figure 5.

**TABLE 3:** Origin, classification, and pharmacological effects of phytochemicals as GSK‐3β inhibitors in the treatment of Alzheimer's disease.

Phytochemicals	Origin	Species	In vivo/in vitro	Model	Dose/time/administration	Change indicators	References
Oxyphylla A	*Alpinia oxyphylla*	Flavonoids	In vitro, in vivo	N2a/APP cells/SAMP8 mice	12–400 μM, 10, 20 mg/kg, 7 days, i.g.	Decrease APP, Aβ_1‐40_, Aβ_1‐42_, Keap1, upregulate p‐Akt (ser473), p‐GSK‐3β (ser9), Nrf2, HO‐1, and NQO1 expression	Bian et al.^145^
Schisandrin B	*Schisandra chinensis*	Polyphenols	In vitro, in vivo	Aβ_25‐35_‐induced SH‐SY5Y cells, APP/PS1 mice	5, 10, 25 μM, 30 mg/kg, 4 weeks, i.g.	Upregulate the expression of p‐GSK‐3β (Ser9) and downregulate the expression of p‐GSK‐3β (Tyr216 and Tyr279)	Hu et al.^149^
1,8‐Cineole	Leaves of eucalyptus trees	Terpenoids	In vitro, in vivo	AGEs induced SH‐SY5Y cell; AGEs induced SD rats	5, 10, and 20 μmol/L, 50, 100, 150 mg/kg, 28 days, ICV	Reduce the abnormal phosphorylation of tau protein at thr205, thr181, and ser396, inhibit BACE‐1 activity, downregulate GSK‐3β activity, reduce Aβ production	An et al.^151^
Quercetin	*Quercus tinctoria*	Flavonoids	In vitro, in vivo	AβO‐induced PHN, AβO‐induced Swiss mice	10, 50, and 100 μmol/L, 300 pmol, 24 h	Increase phosphorylated GSK‐3β S9 levels, impairs GSK‐3β activity	Predes et al.^154^
Morin	*Morus alba*	Flavonoids	In vitro	NA	0, 6.25, 12.5, 25, 50, 100 μM, 24 h	Reduce tau pathology by inhibiting GSK‐3β	Kim et al.^157^
Magnolol	*Magnolia officinalis*	Polyphenols	In vivo	TgCRND8 mice	20, 40 mg/kg, 4 months, i.g.	Increase the ratios of p‐GSK‐3β (Ser9)/GSK‐3β, p‐Akt (Ser473)/Akt, and p‐NF‐κB p65/NF‐κB p65	Xian et al.^159^
Dieckol	*Brown algae*	Polyphenols	In vitro	SweAPP N2a cell	1, 10, and 50 μM, 24 h	Increased the phosphorylation of Akt at Ser473 and GSK‐3β at Ser9, decreased BACE1 protein level and Aβ production	Yoon et al.^162^
Andrographolide	*Andrographis paniculata*	Terpenoids	In vivo	AβPPswe/PS‐1 mice	2.0 mg/kg, 4 weeks, i.p.	Increase the level of the inactive form (phosphorylation of serine‐9) of GSK‐3β, reduce Aβ levels and tau phosphorylation	Serrano et al.^165^
Icariin	*Epimedium sagittatum*	Flavonoids	In vitro	Aβ_25‐35_‐induced PC12 cells	5, 10, and 20 μM, 24 h	Inhibit tau protein hyperphosphorylation at Ser396, Ser404, and Thr205 sites, inhibit the activation of GSK‐3β	Zeng et al.^167^
Linarin	*Mentha arvensis*	Flavonoids	In vitro	Aβ_25‐35_‐induced PC12 cells	0.1, 1.0, and 10 μM	Activate PI3K/Akt, stimulate phosphorylation of GSK‐3β, upregulate the expression of protein Bcl‐2	Lou et al.^169^
Epibrassinolides	*Brassica napus*	Brassinosteroid	In vitro, in vivo	Aβ_42_‐induced PC 12 cells and *Caenorhabditis elegans*	10 μM, 30 μM, 24 h	Increase phosphorylation of GSK‐3β and decrease CDK5 expression, increase β‐catenin translocation	Obakan Yerlikaya et al.^172^
Genipin	*Gardenia jasminoides* Ellis	Flavonoids	In vitro	N2a/SweAPP cells	5, 10, 20, 30, and 40 μM, 24 h	Reduce expression levels of CDK5 and pY216 GSK‐3β, increase expression of SIRT1, p‐LKB1, and p‐AMPK, decrease BACE1 and inhibit Aβ production	Li et al.^174^
Isoorientin	*Sorghum vulgare*	Flavonoids	In vivo	APP/PS1 mice	25, 50 mg/kg, 60 days, i.g.	Increase levels of pSer9‐GSK‐3β, reduce Aβ_42_ levels and Aβ deposition, inhibit the activation of microglia, reduce TNF‐α, IL‐6, and IL‐1β transcripts and decrease the COX‐2 expression, suppress activation and nuclear translocation NF‐κB	Tan et al.^178^
Falcarindiol	*Oenanthe javanica*	Terpenoids	In vitro, in vivo	Glutamate‐induced HT22 cells, diabetic GK rats	0, 10, 20, 50 μM, 15 mg/kg, 24 h, p.o.	Inhibit GSK‐3β in an ATP noncompetitive binding mode	Yoshida et al.^182^
Ginsenoside Rd	*Panax ginseng* Meyer	Ginsenosides	In vivo	APP mice	10 mg/kg, 6 months, i.p.	Depress the expression of GSK‐3β/Tyr216 and CDK5	Li et al.^174^
GP‐17	*Gynostemma pentaphyllum*	Gypenoside	In vitro	Aβ_25‐35_‐induced PC12 cells	2.5, 5, 10, 20 μM	Activate Nrf2, increase the phosphorylation of both Akt and GSK‐3β	Meng et al.^188^
Sulforaphene	Raphani Semen	Isothiocyanates	In vitro, in vivo	LPS‐stimulated BV‐2 cells, STZ‐treated SD rats	0.5–32 μM, 25, 50 mg/kg, 6 weeks, p.o.	Inhibit tau phosphorylation at Thr205, Ser396, and Ser404, increase the ratios of p‐Akt (Ser473)/Akt and p‐GSK‐3β (Ser9)/GSK‐3β, inhibit the production of TNF‐α, IL‐6, increase the release of IL‐10	Yang et al.^75^

Abbreviations: AGEs, advanced glycation end products; AMPK, AMP‐activated protein kinase; Akt, protein kinase B; APP/PS1, amyloid precursor protein/presenilin 1; ATP, adenosine triphosphate; BACE1, β‐site APP cleaving enzyme 1; Bcl‐2, B‐cell lymphoma‐2; CDK5, cyclin‐dependent kinase 5; COX‐2, cyclooxygenase‐2; GK, Goto‐Kakizaki; GP‐17, gypenoside XVII; GSK‐3β, glycogen synthase kinase‐3 beta; HO‐1, heme oxygenase‐1; ICV, intracerebroventricularly injected; i.g., oral gavage; IL, interleukin; i.p., intraperitoneal injections; LKB1, liver kinase B1; LPS, lipopolysaccharide; NQO1, NAD(P)H quinone oxidoreductase 1; NF‐κB, nuclear factor‐kappa B; Nrf2, nuclear factor erythroid 2‐related factor 2; PC12, pheochromocytoma; PHN, primary hippocampal neuron; PI3K/Akt, phosphoinositide 3‐kinase/protein kinase B; SAMP8, senescence‐accelerated mouse prone 8; SIRT1, sirtuin 1; STZ, streptozotocin; SweAPP N2a, Swedish mutant amyloid precursor protein overexpressed N2a; TNF‐α, tumor necrosis factor‐α.

Oxyphylla A is a natural compound derived from the plant *Alpinia oxyphylla*. Oxyphylla A belongs to a class of compounds known as flavonoids. Oxyphylla A has attracted attention owing to its potential pharmacological properties and biological activities.^142^ Oxyphylla A exhibits various biological effects, including antioxidant, anti‐inflammatory, and anticancer activities.^143^, ^144^ In N2a/APP cells and SAMP8 mice (an in vitro and in vivo AD model), oxyphylla A reduced the protein expression levels of APP and Aβ by upregulating p‐GSK‐3β (ser9) and activated the Nrf2/Keap1/HO‐1 pathway by inhibiting GSK‐3β.^145^

Schisandrin B is a natural compound derived from *Schisandra chinensis*. Schisandrin B is classified as a lignan, a polyphenolic compound found in various plants. Schisandrin B has been studied for its potential health benefits and pharmacological properties.^146^ Schisandrin B exhibits antioxidant, anti‐inflammatory, and hepatoprotective effects. Schisandrin B removes free radicals and reduces oxidative stress.^147^ Additionally, schisandrin B has demonstrated potential in protecting against neurodegenerative diseases by modulating neurotransmitter levels and reducing oxidative stress in the brain.^148^ Besides, schisandrin B upregulated p‐GSK‐3β (Ser9) expression and downregulated p‐GSK‐3β (Tyr216) and p‐GSK‐3β (Tyr279) expressions in in vitro and in vivo AD models. Schisandrin B can attenuate Aβ‐induced cellular damage and cognitive impairment in AD mice and may act as a potential selective ATP‐competitive GSK‐3β inhibitor, further affecting its anti‐AD effect.^149^

1,8‐Cineole, also known as eucalyptol, is a natural organic compound found in various plants, most notably in the leaves of eucalyptus trees. 1,8‐Cineole may have neuroprotective properties and could be beneficial in AD prevention or treatment.^150^ 1,8‐Cineole possesses antioxidant and anti‐inflammatory effects, which are important factors in neuroprotection. In the advanced glycation end products induced AD model in vitro and in vivo, 1,8‐cineole can reduce abnormal tau protein phosphorylation at thr205, thr181, and ser396 by inhibiting BACE‐1 activity, downregulating GSK‐3β activity, and reducing Aβ production.^151^

Quercetin is a naturally occurring flavonoid compound found in various fruits, vegetables, and plants. Quercetin is known for its antioxidant and anti‐inflammatory properties, including potential health benefits. Quercetin may have neuroprotective properties that may be beneficial for AD prevention or treatment.^152^ Quercetin has antioxidant and anti‐inflammatory effects, which are important factors for neuroprotection. In addition, quercetin has an inhibitory effect on enzymes involved in Ach breakdown, and by inhibiting AchE, quercetin may increase Ach levels in the brain and possibly improve cognitive function.^153^ In Aβ oligomers (AβO)‐induced Swiss mice, quercitrin enhanced Wnt signaling by reducing GSK‐3 activity and increasing phosphorylated GSK‐3β (Ser9) level to prevent hippocampal synaptic loss and AβO‐mediated memory impairment.^154^

Morin is a natural flavonoid compound found in various plants, including *Morus alba* (white mulberry) and *Maclura pomifera* (osage orange), certain fruits, vegetables, and herbs.^155^ Morin is known for its antioxidant and anti‐inflammatory properties, including potential health benefits. Morin can improve cognitive function, reduce oxidative stress, inhibit cholinesterase activity, and protect against Aβ peptide‐induced neurotoxicity in the brain.^156^ Morin is a GSK‐3β inhibitor that reduces tau pathology in vivo and in vitro, and morin inhibits GSK‐3β by binding to the ATP‐binding pocket, suggesting that morin may serve as a functional food for AD treatment.^157^

Magnolol, an important natural neolignan, is the main active ingredient of *Magnolia officinalis* bark, which has therapeutic effects. Magnolol has an anti‐AD effect in experimental AD model.^158^ Magnolol significantly reduces the neurotoxicity induced by Aβ by inhibiting the increase of intracellular calcium, ROS production, caspase‐3 activity, and inflammatory response and promoting Aβ phagocytosis and degradation. Besides, magnolol treatment significantly increased the protein expression ratios of p‐GSK‐3β (Ser9)/GSK‐3β and p‐Akt (Ser473)/Akt in the brain tissue of AD mice, decreased the protein levels of Aβ_40_ and Aβ_42_, and therefore significantly improved the cognitive deficits of TgCRND8 mice.^159^

Dieckol is a specific phlorotannin compound found in brown algae, such as *Ecklonia cava* and *stolonifera*. Dieckol is one of the major phlorotannins found in these algae and has attracted attention owing to its potential health benefits.^160^ Dieckol has been studied for its antioxidant, anti‐inflammatory, antimicrobial, and anticancer properties. Dieckol decreased the autoaggregation of Aβ_25–35_ with an IC_50_ value of 7.9 ± 0.2.^161^ Dieckol increased Akt phosphorylation at Ser473 and GSK‐3β at Ser9, indicating that dieckol induced Akt activation and GSK‐3β phosphorylation. Additionally, dieckol promoted the PI3K/Akt signaling pathway, which in turn inactivated GSK‐3β, resulting in reduced Aβ levels.^162^

Andrographolide, also known as the “King of Bitters,” is a bioactive diterpenoid lactone and the main active component responsible for the medicinal properties of *Andrographis paniculata*.^163^ Andrographolide has been used in traditional Chinese medicine for various health benefits. Andrographolide possesses neuroprotective properties by reducing oxidative stress, inflammation, and neurotoxicity, which are implicated in AD development and progression.^164^ Besides, andrographolide protects hippocampal neurons from Aβ oligomers in AD mice, and the mechanism may be related to GSK‐3β inhibition by increasing the level of the inactive form (serine‐9 phosphorylation) of GSK‐3β. Moreover, andrographolide reduces Aβ levels, including tau phosphorylation around Aβ oligomers. Andrographolide restores the spatial memory function associated with the protection of synaptic plasticity and synaptic proteins, suggesting that andrographolide can be used as a potential preventive treatment for AD.^165^

Icariin is a natural compound found in several plants, most notably in the herb *Epimedium sagittatum*, commonly known as “Horny Goat Weed.” Icariin belongs to a class of compounds called flavonoids, specifically known as prenylated flavonoids. Icariin has gained attention for its potential anti‐inflammatory, antioxidant activity, and neuroprotective effects.^166^ Zeng et al. indicated that icariin could reduce Aβ_25–35_‐induced tau protein hyperphosphorylation and promote neuronal cell survival. Icariin activates the PI3K/Akt signaling pathway, thereby increasing the GSK‐3β phosphorylation level at the Ser9 site. Icariin may be one of the potential drugs for AD and other neuronal degenerative diseases related to tau pathology.^167^

Linarin is a natural compound belonging to the flavonoid class. Linarin is also known as locubin‐7‐O‐glucoside and is commonly found in various plants, including *Mentha arvensis* and *Buddleja davidii*. Linarin has neuroprotective effects by reducing oxidative stress and inflammation, inhibiting Aβ formation and aggregation, including AchE activity.^168^ pSer9‐related GSK‐3β inhibition may be one of the mechanisms by which linarin protects neuronal cells against apoptosis induced by Aβ_25–35_. Linarin activated PI3K/Akt and stimulated GSK‐3β phosphorylation. Moreover, the expression of the antiapoptotic protein Bcl‐2 was also increased by linarin.^169^

Epibrassinolides, a member of the brassinolide family of plant growth hormones that are structurally similar to mammalian steroid hormones, induce apoptotic cell death in mammalian cancer cells.^170^ Epibrassinolides attenuated dopaminergic neurons from 1‐methyl‐4‐phenylpyridine‐induced oxidative stress and subsequent apoptosis.^171^ Epibrassinolides protected cells from death and increased the survival rates of *Caenorhabditis elegans* strains induced by tau hyperphosphorylation. Moreover, epibrassinolides increased GSK‐3β phosphorylation and decreased CDK5 expression in tauopathy model PC12 cells, suggesting that epibrassinolides have neuroprotective effects in vitro and in *C. elegans* model by inhibiting GSK‐3β Ser9 phosphorylation.^172^

Genipin is a natural compound derived from the *Gardenia jasminoides* Ellis that has been studied for its potential therapeutic effects on neurodegenerative diseases. Genipin has antioxidant, anti‐inflammatory, and antiapoptotic activities, which protect neurons from damage and promote their survival.^173^ Specifically, genipin binds to tau proteins and prevents tau fibers from forming. Genipin may inhibit tau phosphorylation by downregulating CDK5 and GSK‐3β expressions. In addition, genipin reduced Aβ production by inhibiting BACE1 expression, suggesting that genipin may be developed as an effective therapeutic complement or potential nutritional supplement to prevent AD.^174^

Isoorientin is a natural compound found in various plants. Isoorientin is found in Sorghum (*Sorghum vulgare*), *Perilla frutescens*, eggplant (*Solanum melongena*), and other plants. Isoorientin exhibits various activities beneficial to the nervous system. First, isoorientin has an antioxidant effect, which can neutralize free radical production and reduce oxidative stress damage on nerve cells.^175^ Second, isoorientin has anti‐inflammatory effects, which can inhibit the inflammatory response and release of inflammatory factors, thereby reducing neuroinflammation occurrence and progression.^176^ In addition, isoorientin has neuroprotective effects, promoting nerve cell survival and increasing nerve cell resistance to damage.^177^ Tan et al. found that isoorientin can improve spatial learning and memory deficits in APP/PS1 mice. Mechanism studies suggested that isoorientin may play a neuroprotective role through multiple pathways. Isoorientin significantly increased pSer9‐GSK‐3β level, inhibiting tau protein phosphorylation and NFT formation. Moreover, isoorientin reduced Aβ_42_ levels and Aβ deposition and inhibited neuroinflammatory response.^178^

Falcarindiol is a polycyclic diterpenoid, which is mainly found in Japanese parsley (*Oenanthe javanica*). Falcarindiol has been extensively studied and has shown various biological activities and pharmacological potential. Falcarindiol has antioxidant, anti‐inflammatory, and antibacterial effects.^179^, ^180^ Falcarindiol may have neuroprotective properties and could potentially be beneficial in AD management.^181^ Yoshida et al. indicated that falcarindiol inhibited G6Pase gene expression in rat hepatoma cells and protected mouse neuroblastoma cells from glutamate‐induced oxidative cell death. Falcarindiol may interact with primed phosphoric acid binding pocket and Gln89‐Asn95 loops on GSK‐3β via hydrogen or covalent bonding to inhibit GSK‐3β in an ATP noncompetitive binding mode and reduce the risk of brain insulin‐resistant AD.^182^

Ginsenoside Rd has been the subject of scientific research owing to its potential therapeutic properties. Ginsenoside Rd has various pharmacological effects, including neuroprotective, anti‐inflammatory, antioxidant, and cardiovascular protective activities.^183^ Regarding neuroprotection, ginsenoside Rd has shown potential in protecting against neurodegenerative diseases, including AD. Ginsenoside Rd may be a promising drug for AD treatment and is associated with various pathological changes in AD, including Aβ deposition and tau hyperphosphorylation.^184^ In the study of APP transgenic mice pretreated with ginsenoside Rd ameliorated learning and memory ability. Li et al.^185^ demonstrated that ginsenoside Rd effectively inhibited hyperphosphorylated tau (S199/202, S396, and S404) protein production and deposition by depressing GSK‐3β/Tyr216 expression, suggesting the potential therapeutic effect of ginsenoside Rd in early AD.

Gypenoside XVII (GP‐17) is a bioactive compound known as gypenoside. GP‐17 is derived from *Gynostemma pentaphyllum*, a traditional Chinese herb commonly known as jiaogulan or southern ginseng.^186^ GP‐17 has been the subject of scientific research owing to its potential health benefits. GP‐17 possesses various pharmacological properties, including antioxidant, anti‐inflammatory, anticancer, and cardioprotective activities.^187^ GSK‐3β inhibition is involved in GP‐17‐mediated neuroprotection. Meng et al. indicated that Akt and GSK‐3β phosphorylations mediated by GP‐17 were effectively promoted; furthermore, GP‐17 also attenuated Aβ_25–35_‐induced multiple pathogenic mechanisms, including apoptosis, autophagy, and oxidative stress in PC12 cells, providing a novel insight into understanding the mechanism for neuroprotective GP‐17 effects.^188^

Sulforaphene, a natural compound found in Raphani Semen, has shown potential in the field of AD. Sulforaphene may have neuroprotective effects and could be beneficial for individuals with AD. Sulforaphene exhibits antioxidant activity, which can help reduce oxidative stress and protect neurons from damage caused by free radicals.^189^ Sulforaphene has demonstrated anti‐inflammatory effects. By suppressing inflammation, sulforaphene may help reduce the harmful effects of inflammation on brain cells. Additionally, sulforaphene can potentially promote the clearance of Aβ plaques.^190^ Further study showed that sulforaphene significantly inhibited tau phosphorylation at Thr205, Ser396, and Ser404, and the ratios of p‐Akt (Ser473)/Akt and p‐GSK‐3β (Ser9)/GSK‐3β in the hippocampus of streptozotocin (STZ)‐treated rats were also increased. Sulforaphene treatment significantly inhibited the production of tumor necrosis factor‐α (TNF‐α) and interleukin‐6 (IL‐6) and increased the release of IL‐10 in STZ‐treated rats, suggesting that sulforaphene is a promising neuroprotective agent with neuroprotective effects on STZ‐induced cognitive deficits.^75^

## 7 IN SILICO MOLECULAR DOCKING OF PHYTOCHEMICALS TO GSK‐3β

**FIGURE 6:**
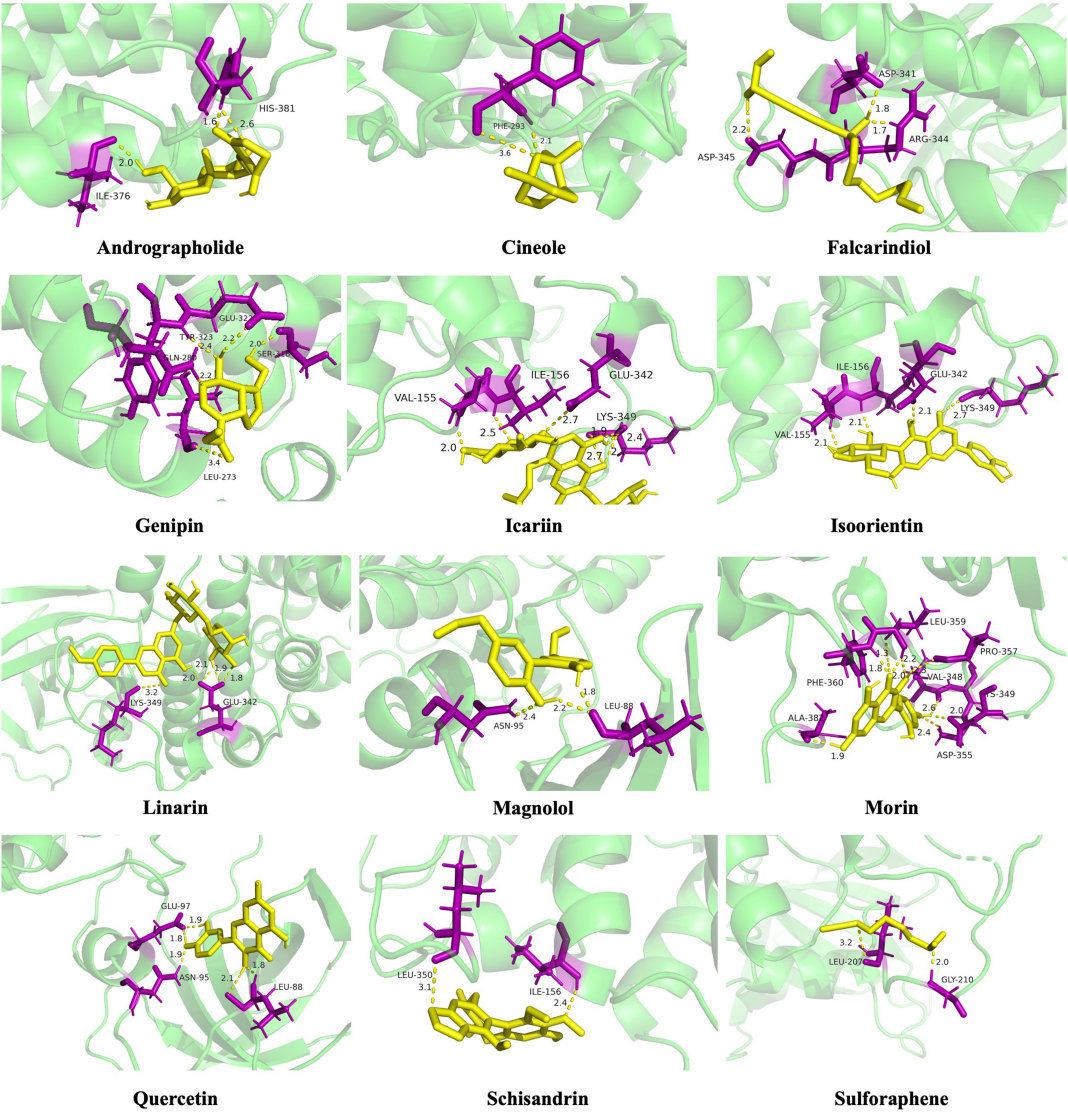
Defined molecular docking of phytochemicals to GSK‐3β. The residues bound to compounds by hydrogen bonds are shown in bold.

Natural compounds from herbs and nutraceuticals may provide valuable chemical scaffolds for developing new drugs for AD treatment (as shown in Table 3). Since GSK‐3β‐centered Aβ deposition and tau hyperphosphorylation play critical roles in AD pathogenesis, natural compounds from herbs and nutraceuticals may play a therapeutic role by inhibiting GSK‐3β. This can be illustrated using molecular docking analysis. Table 4 shows the binding energy and pKi values of the above phytochemicals. Visualization of the combination with GSK‐3β is shown in Figure 6.

**TABLE 4:** Defined molecular docking of phytochemicals to GSK‐3β.

Phytochemicals	Lowest energy of docking (kcal/mol)	pKi
Andrographolide	−4.89	261.46 μM
Cineole	−4.45	551.75 μM
Falcarindiol	−2.58	12.74 μM
Genipin	−3.94	1.29 mM
Icariin	−2.35	18.87 mM
Linarin	−3.25	4.15 mM
Magnolol	−4.32	686.73 μM
Morin	−5.27	136.22 μM
Quercetin	−4.64	396.49 μM
Schisandrin	−4.1	979.67 μM
Sulforaphene	−4.5	506.2 μM

According to the results of this study, genipin, icariin, and linarin could bind to the GSK‐3β active sites at the nanomolar scale. Meanwhile, this suggests that these phytochemicals can be considered effective inhibitors for the enzyme. Furthermore, Asn‐95, Glu‐342, and Leu‐88 were the most important amino acids involved in GSK‐3β inhibition.

## 8 CONCLUSIONS

Owing to the incomplete understanding of AD pathogenesis, no effective and safe method to prevent or treat AD exists. The aging population worldwide has led to an increase in AD incidence. This makes research in this area even more urgent. Senile plaques caused by abnormal aggregation of Aβ and NFTs related to tau hyperphosphorylation, which are the main pathological changes in AD, are currently the focus of research in AD treatment. However, drug development, including Aβ monoclonal antibody, is challenging and far from reaching the expected effect. The advantage of natural compounds from herbs and nutraceuticals lies in their safety, multitarget, and multimechanism pharmacological properties, which have gradually been accepted as important sources of new drug development. However, the poor bioavailability of phytochemicals and difficulty crossing the BBB limit their application. Research on phytochemical derivatives can improve the bioavailability and reduce the toxicity of phytochemicals without changing or optimizing their pharmacological properties, which brings hope for the early clinical application of phytochemicals.

Natural compounds from herbs and nutraceuticals effectively inhibit GSK‐3β against major pathological changes in AD, which provides novel strategies for developing drugs for AD treatment. Natural compounds from herbs and nutraceuticals can provide chemical scaffolds that act as lead compounds for producing derivatives with improved pharmacological properties.

## AUTHOR CONTRIBUTION

Zheng Zhao – Department of Emergency Medicine, Shengjing Hospital of China Medical University, Shenyang, Liaoning, China. Ye Yuan – Department of Neurosurgery, Shengjing Hospital of China Medical University, Shenyang, Liaoning, China. Shuang Li – Department of Emergency Medicine, Shengjing Hospital of China Medical University, Shenyang, Liaoning, China. Xiaofeng Wang – Department of Emergency Medicine, Shengjing Hospital of China Medical University, Shenyang, Liaoning, China. Xue Yang – Department of Neurology, Shengjing Hospital of China Medical University, No. 36 Sanhao street, Heping District, Shenyang 110000, Liaoning, China; https://orcid.org/0009‐0008‐4182‐0140; Phone: +86‐18940253551; Email: yangx@sj-hospital.org.

## CONFLICT OF INTEREST STATEMENT

We confirm that there are no known conflicts of interest associated with this publication and there has been no significant financial support for this work that could have influenced its outcome.

## Data Availability

No data were used for the research described in the article.
